# Myocardium-derived conditioned medium improves left ventricular function in rodent acute myocardial infarction

**DOI:** 10.1186/1479-5876-9-11

**Published:** 2011-01-18

**Authors:** Steve Leu, Ying-Hsien Kao, Cheuk-Kwan Sun, Yu-Chun Lin, Tzu-Hsien Tsai, Li-Teh Chang, Sarah Chua, Kuo-Ho Yeh, Chiung-Jen Wu, Morgan Fu, Hon-Kan Yip

**Affiliations:** 1 Division of Cardiology, Department of Internal Medicine, Chang Gung Memorial Hospital - Kaohsiung Medical Center, Chang Gung University College of Medicine, Kaohsiung, Taiwan; 2 Center for Translational Research in Biomedical Sciences, Chang Gung Memorial Hospital - Kaohsiung Medical Center, Chang Gung University College of Medicine, Kaohsiung, Taiwan; 3 Department of Medical Research, E-DA Hospital, I-Shou University, Kaohsiung, Taiwan; 4 Division of General Surgery, Department of Surgery, Chang Gung Memorial Hospital - Kaohsiung Medical Center, Chang Gung University College of Medicine, Kaohsiung, Taiwan; 5 Basic Science, Nursing Department, Meiho University, Pingtung, Taiwan

## Abstract

**Background:**

We investigated whether myocardium-derived conditioned medium (MDCM) is effective in preserving left ventricular (LV) function in a rat acute myocardial infarction (AMI) model.

**Methods:**

Adult male Sprague-Dawley (SD) rats (n = 36) randomized to receive either left coronary artery ligation (AMI induction) or thoracotomy only (sham procedure) were grouped as follows (n = 6 per group): Group I, II, and III were sham-controls treated by fresh medium, normal rat MDCM, and infarct-related MDCM, respectively. Group IV, V, and VI were AMI rats treated by fresh medium, normal MDCM, and infarct-related MDCM, respectively. Either 75 μL MDCM or fresh medium was administered into infarct myocardium, followed by intravenous injection (3 mL) at postoperative 1, 12, and 24 h.

**Results:**

In vitro studies showed higher phosphorylated MMP-2 and MMP-9, but lower α-smooth muscle actin and collagen expressions in neonatal cardiac fibroblasts treated with MDCM compared with those in the cardiac fibroblasts treated with fresh medium (all p < 0.05). Sirius-red staining showed larger collagen deposition area in LV myocardium in Group IV than in other groups (all p < 0.05). Stromal cell-derived factor-1α and CXCR4 protein expressions were higher in Group VI than in other groups (all p < 0.05). The number of von Willebrand factor- and BrdU-positive cells and small vessels in LV myocardium as well as 90-day LV ejection fraction were higher, whereas oxidative stress was lower in Group VI than in Group IV and Group V (all p < 0.05).

**Conclusion:**

MDCM therapy reduced cardiac fibrosis and oxidative stress, enhanced angiogenesis, and preserved 90-day LV function in a rat AMI model.

## Background

Although transplantation of a variety of stem cells has been reported to be beneficial in improving infarct- and ischemia-related LV dysfunction [1-5], the underlying mechanisms are still poorly understood [3-5]. It has been proposed that implanted mesenchymal stem cells (MSCs) differentiated into functional cardiomyocytes to replace the lost myocardium, thereby improving heart function [6]. However, accumulating evidence has shown that only a few implanted stem cells subsequently express myogenic cell-like phenotype in ischemic zone [3-5,7]. Direct cellular participation, therefore, seems an unlikely explanation for the improvement in LV function after cell therapy. In contrast, growing data [4,5,8-11] support that angiogenesis, trophic and paracrine (i.e. cytokine and chemokine) effects, as well as stem cell homing appear to be possible mechanisms underlying the improved heart function following stem cell treatment.

Matrix metalloproteinases (MMPs) participate in reducing cardiac remodeling through regulating the degradation of extracellular matrix (ECM) and fibrosis after acute myocardial infarction (AMI) [12,13]. Cardiac fibroblasts (CFBs), which constitute 60-70% of cells in the human heart, have distinctive properties of secreting cytokines and chemokines in response to various stimuli such as ischemia or mechanical stress to the heart [12]. In addition, CFBs have been reported to have the ability of secreting MMPs in response to the stimulation from implanted mesenchymal stem cells in ischemia area [13]. Furthermore, abundant data from both clinical observational and experimental studies have revealed that ischemic preconditioning can salvage myocardium in the settings of ischemia-reperfusion injury and AMI [14-17]. Additionally, enhancement of neovascularization and collateral circulation in ischemic area, which has been observed in AMI patients with ischemic preconditioning [18,19], has also been reported to contribute to better prognostic outcome [19,20]. These findings [14-20] raise the hypothesis that ischemic preconditioning may participate in enhancing the secretion of chemokines/cytokines which are essential for angiogenesis/neovascularization.

In the present study, therefore, we first prepared myocardial infarct-related myocardium-derived conditioned medium (MDCM) to mimic the setting of ischemic preconditioning. We further tested the hypothesis that the conditioned medium from in vitro culturing of different cellular components of the heart including cardiomyocytes, endothelial cells, and CFBs may contain SDF-1α and vascular endothelial growth factor (VEGF), two key angiogenesis-related mediators, and other cytokines. The therapeutic impact of the conditioned medium on cardiac remodeling, heart function, cardiac fibrosis, and angiogenesis was also investigated in vivo in a rat AMI model.

## Methods

### Ethics

All experimental animal procedures were approved by the Institute of Animal Care and Use Committee at our hospital and performed in accordance with the Guide for the Care and Use of Laboratory Animals (NIH publication No. 85-23, National Academy Press, Washington, DC, USA, revised 1996).

### Animals, Protocol and Procedure

Experimental procedures were performed in pathogen-free, adult male Sprague-Dawley (SD) rats, weighing 275-300 g (Charles River Technology, BioLASCO Taiwan Co., Ltd., Taiwan). The detailed procedure was based on our previous report [4]. Briefly, SD rats were anesthetized by intraperitoneal injections of chloral hydrate (35 mg/kg). The rat was placed in a supine position on a warming pad at 37°C after being shaved on the chest and then intubated with positive-pressure ventilation (180 mL/min) with room air using a Small Animal Ventilator (SAR-830/A, CWE, Inc., USA). Under sterile conditions, the heart was exposed via a left thoracotomy at the level of 5^th ^intercostal space.

Sham-operated control rats (n = 18) that only received thoracotomy without left coronary artery ligation (LCAL) were further divided into three groups (n = 6 per group): Group I [Sham controls with 75 μl of fresh medium (DMEM plus 10% of fetal bovine serum)] infused into LV anterior wall at six different sites); Group II [Sham controls with 75 μl of normal rat myocardium-derived conditioned medium (MDCM) injected into LV anterior wall]; Group III (Sham controls with 75 μl of infarct-related MDCM injected into LV anterior wall).

**Figure 1:**
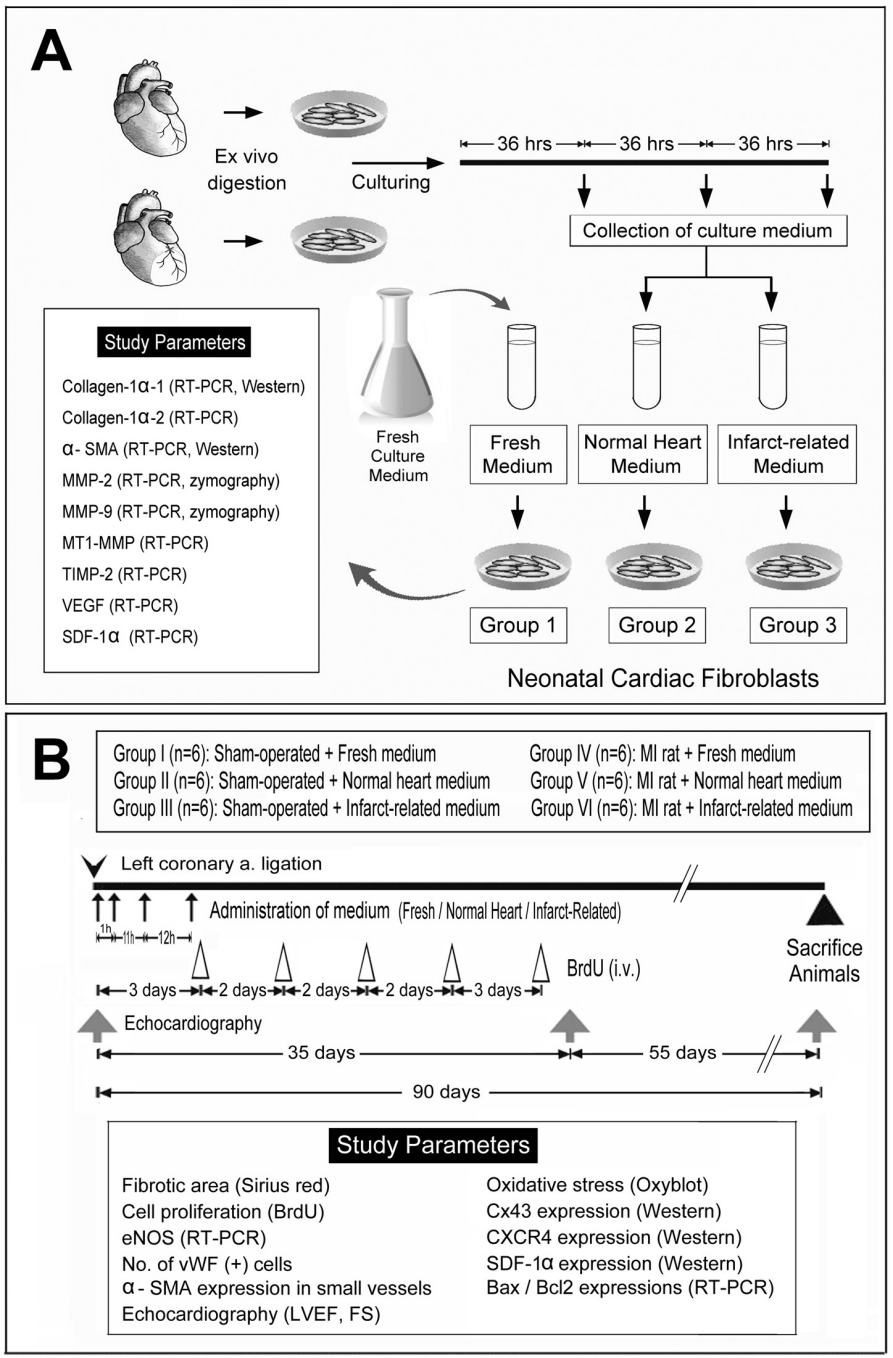
**Detailed protocol and procedure**. Schematic illustration of the detailed protocol on preparative procedure of conditioned media and treatment courses as well as in vitro and ex vivo molecular-cellular studies.

AMI induction (n = 18) was performed through left coronary artery ligation (LCAL) 2 mm below the left atrium with a 7-0 prolene suture. Regional myocardial ischemia was confirmed through the observation of a rapid discoloration over the anterior surface of the LV together with the development of akinesia and dilatation over the at-risk area. These rats were further assigned into three groups (n = 6 per group): Group IV (AMI induction plus 75 μl of fresh medium injected into LV anterior wall at six different sites); Group V (AMI induction plus 75 μl of normal rat MDCM injected into LV anterior wall), and Group VI (AMI induction plus 75 μl of infarct-related MDCM injected into LV anterior wall). Both fresh and conditioned media were injected into the ischemic area of LV wall 30 minutes after AMI induction. Three milliliters of either MDCM or fresh medium was intravenously administered at postoperative 1, 12, and 24 h for individual Group of rats (Figure 1B).

To determine the impact of conditioned medium therapy on collagen deposition in infarct area using Sirius red staining, sixteen additional adult male SD rats having received the same procedure and treatment as Groups I, IV, V, and VI (n = 4 in each group) were also included in this study.

### Preparation of Conditioned Media for Infusion

Twelve extra SD rats, including six normal rats and six rats 72 h after LCAL were utilized for media preparation (Figure 1A). Each rat was euthanized by an overdose of intraperitoneal sodium pentobarbital and the heart was then removed immediately after opening the chest wall and attached to the perfusion pump. All procedures and the ingredients of the perfusion solutions were in accordance with previously reported protocols [21]. Briefly, the adult male SD rats (~350 g) were euthanized by an intraperitoneal injection of sodium pentobarbital (100 mg/kg). Cell component of myocardium was isolated by a modified method of Mitra and Morad. The heart was removed and perfused retrogradely at 37°C for 5 minutes with Ca^2+^-free Tyrode solution containing (in mM) 137 NaCl, 5 KCl, 1 MgCl_2_, 10 D-glucose, and 10 NaHEPES (HEPES neutralized to pH 7.4 with NaOH). This was followed by recirculation of the same solution containing (U/ml) 300 collagenase (type I) and 1 protease (type XIV) for 10 minutes and then perfusion with enzyme-free Tyrode solution containing 0.2 mM CaCl_2 _for a further 5 minutes to stop enzymatic digestion. The ventricles were cut radially, and the cells were dispersed at room temperature for experiments within 8 h of isolation. The myocardium components of each rat, which included cardiomyocytes, endothelial cells, and CFBs, were collectively isolated and cultured in DMEM culture medium [in 50 mL of 150 cm^2 ^flask (1.0 × 10^6 ^cells per mL culture medium)]. The supernatants were collected at 36 h after cell culture and then stored at -20°C for future use. These supernatants were defined as 1) Normal (without AMI) MDCM and 2) Infarct-related MDCM.

### Definition of Conditioned Medium

The culture media utilized in the current study were categorized into (1) Fresh medium (G1); (2) Normal MDCM derived from cardiac cellular components of normal rat hearts (G2); (3) Infarct-related MDCM derived from cardiac cellular components of infarcted hearts (G3). To investigate the concentration-dependent impact, two concentrations (i.e. 10% and 20%) of G2 and G3 media were adopted in the current study. The 10% G2 medium was prepared by mixing 10% of G2 with 90% of G1, while the 20% G2 medium was prepared by mixing 20% of G2 with 80% of G1. Similarly, the 10% and 20% G3 media were prepared by mixing 10% and 20% of G3 with 90% and 80% of G1, respectively.

### Functional Assessment by Echocardiography

Transthoracic echocardiography was performed in each group prior to and on day 90 after AMI induction with the anesthetized rats in a supine position by an animal cardiologist blinded to the design of the experiment using a commercially available echocardiographic system (UF-750XT) equipped with a 8-MHz linear-array transducer for animals (FUKUDA Denshi Co. Hongo, Bunkyo-Ku, Tokyo, Japan). M-mode tracings of LV were obtained with the heart being imaged in 2-dimensional mode in short-axis at the level of the papillary muscle. Left ventricular internal dimensions [end-systolic diameter (ESD) and end-diastolic diameter (EDD)] were measured according to the American Society of Echocardiography leading-edge method using at least three consecutives cardiac cycles. The LV ejection fraction (LVEF) was calculated as follows: LVEF (%) = [(LVEDD^3^-LVEDS^3^)/LVEDD^3^] × 100

### Preparation of Neonatal Cardiac Fibroblasts and Grouping (Figure 1)

Three-day-old newborn SD rats were euthanized by an overdose of intraperitoneal sodium pentobarbital. The hearts were removed after opening the chest wall and cut into pieces, followed by further lyses in enzymatic digestive solution [50 mL PBS buffer containing 0.07 g collagenase IV (Sigma), 14 mg protease XIV (Sigma) and 0.09 g glucose]. Finally, the CFBs were collected and co-cultured with conditioned media.

The harvested CFBs (Figure 1A) were then divided into three groups according to the culture medium in which they were incubated: Group 1 (5.0 × 10^5 ^CFBs cultured in fresh medium for 48 h), Group 2 (5.0 × 10^5 ^CFBs co-cultured with 10% and 20% of normal MDCM for 48 h, respectively), and Group 3 (5.0 × 10^5 ^CFBs co-cultured with 10% and 20% of infarct-related MDCM for 48 h, respectively).

### Cellular Proliferation Test

To evaluate whether MDCM treatment promotes cellular proliferation in the infarct area, 5-bromodeoxyuridine (BrdU) was intravenously given in Groups I, IV, and VI animals on days 3, 5, 7, 9, and 12 after acute AMI induction for labeling the proliferating cells.

### Specimen Collection

Rats in each group were euthanized on day 90 after AMI induction, and heart in each rat was rapidly removed and immersed in cold saline. For immunohistofluorescence (IHF) study, the heart tissue was rinsed with PBS, embedded in OCT compound (Tissue-Tek, Sakura, Netherlands) and snap-frozen in liquid nitrogen before being stored at -80°C. For immunohistochemical (IHC) staining, heart tissue was fixed in 4% formaldehyde and embedded in paraffin.

### IHC Staining

Cardiac cross-sections were collected in the sixteen additional rats in Groups I, IV, V, and IV (n = 4 per group). To analyze the extent of collagen synthesis and deposition, three cardiac paraffin sections (6 μm) at 3 mm intervals were stained with picro-Sirius red (1% Sirius red in saturated picric acid solution) for one hour at room temperature using standard methods. The sections were then washed twice with 0.5% acetic acid. After dehydration in 100% ethanol thrice, the sections were cleaned with xylene and mounted in a resinous medium. Ten low power fields (×10) of each section were used to identify Sirius red-positive area on each section. Image-pro plus 6.1 software (Media Cybernetics, Inc., Bethesda, MD, USA) was used to calculate the total cross-sectional area of left ventricle and the total area of Sirius red-positive staining. The mean area of collagen deposition (A) was obtained by summation of Sirius red-positive areas on each section divided by the total numbers of sections. In addition, the mean cross-sectional area (B) of left ventricle was obtained by dividing the sum of all cross sectional areas with the total number of sections examined. Finally, the percentage change in area of collagen deposition was obtained by dividing (A) with (B), followed by multiplication by 100%.

IHC of blood vessels was performed by incubating the tissue sections with an anti-α-SMA (1:400) primary antibody at room temperature for 1 h, followed by washing with PBS thrice. Ten minutes after the addition of the anti-mouse-HRP conjugated secondary antibody, the tissue sections were washed with PBS thrice again. The 3,3' diaminobenzidine (DAB) (0.7 gm/tablet) (Sigma) was then added, followed by washing with PBS thrice after one minute. Finally, hematoxylin was added as a counter-stain for nuclei, followed by washing twice with PBS after one minute. Three sections of LV myocardium were analyzed in each rat. For quantification, three randomly selected HPFs (×100) were analyzed in each section. The mean number per HPF for each animal was then determined by summation of all numbers divided by 9.

### Western Blot Analysis for Connexin (Cx)43, CXCR4, Stromal Cell-Derived Factor (SDF)-1α, and Oxidative Stress Reaction in LV Myocardium

Equal amounts (10-30 mg) of protein extracts from remote viable LV myocardium were loaded and separated by SDS-PAGE using 8-10% acrylamide gradients. Following electrophoresis, the separated proteins were transferred electrophoretically to a polyvinylidene difluoride (PVDF) membrane (Amersham Biosciences). Nonspecific proteins were blocked by incubating the membrane in blocking buffer (5% nonfat dry milk in T-TBS containing 0.05% Tween 20) overnight. The membranes were incubated with the indicated primary antibodies (Cx43, 1:1000, Chemicon; CXCR4, 1:1000, Abcam; SDF-1, 1:1000, Cell Signaling; Actin, 1:10000, Chemicon) for 1 h at room temperature for Cx43 and CXCR4 and overnight at 4°C for SDF-1, respectively. Horseradish peroxidase-conjugated anti-mouse immunoglobulin IgG (1:2000, Amersham Biosciences) was applied as the second antibody for Cx43 for 1 h at room temperature; Horseradish peroxidase-conjugated anti-rabbit immunoglobulin IgG (1:2000, Cell Signaling) was applied as the secondary antibody for 1 h for CXCR4 and 45 minutes for SDF-1 at room temperature. The washing procedure was repeated eight times within 1 h.

The Oxyblot Oxidized Protein Detection Kit was purchased from Chemicon (S7150). The oxyblot procedure was performed according to our recent study [5]. The procedure of 2,4-dinitrophenylhydrazine (DNPH) derivatization was carried out on 6 μg of protein for 15 minutes according to manufacturer's instructions. One-dimensional electrophoresis was carried out on 12% SDS/polyacrylamide gel after DNPH derivatization. Proteins were transferred to nitrocellulose membranes which were then incubated in the primary antibody solution (anti-DNP 1: 150) for 2 h, followed by incubation with second antibody solution (1:300) for 1 h at room temperature. The washing procedure was repeated eight times within 40 minutes.

Immunoreactive bands were visualized by enhanced chemiluminescence (ECL; Amersham Biosciences) which was then exposed to Biomax L film (Kodak). For quantification, ECL signals were digitized using Labwork software (UVP). For oxyblot protein analysis, a standard control was loaded on each gel.

### Real-Time Quantitative PCR Analysis

Real-time polymerase chain reaction (RT-PCR) was conducted using LightCycler TaqMan Master (Roche, Germany) in a single capillary tube according to the manufacturer's guidelines for individual component concentrations as we previously reported [5]. Forward and reverse primers were each designed based on individual exons of the target gene sequence to avoid amplifying genomic DNA.

During PCR, the probe was hybridized to its complementary single-strand DNA sequence within the PCR target. As amplification occurred, the probe was degraded due to the exonuclease activity of Taq DNA polymerase, thereby separating the quencher from reporter dye during extension. During the entire amplification cycle, light emission increased exponentially. A positive result was determined by identifying the threshold cycle value at which reporter dye emission appeared above background.

### Zymography Analysis Amplification

For zymography, supernatants from cultured neonatal cardiac fibroblasts (CFBs) (Group 1, 10% and 20% of Groups 2 and 3) were collected and centrifuged (500 g, 5 min) to remove cells and debris. Protein extract was electrophoresed in 8% SDS-PAGE containing 0.1% gelatin. After migration and washing, gels were incubated (16 h, 37°C) in activation buffer (50 mM Tris-base at pH 7.5, 5 mM CaCl_2_, 0.02% NaN_3_, and 1 μM ZnCl_2_). Gels were stained with Coomassie staining solution (0.5% Coomassie, 50% MeOH, 10% acetic acid, and 40% H_2_O) for 90 minutes, followed by destaining (0.5% Coomassie, 50% MeOH, 10% acetic acid, and 40% H_2_O). Quantification of Western blot and zymography was performed with densitometry (TotalLab v1.10, Nonlinear Dynamics; Durham, NC, http://www.nonlinear.com).

### Statistical Analysis

Data were expressed as mean values (mean ± SD). The significance of differences between two groups was evaluated with *t*-test. The significance of differences among the groups was evaluated using analysis of variance followed by Bonferroni multiple-comparison post hoc test. Statistical analyses were performed using SAS statistical software for Windows version 8.2 (SAS institute, Cary, NC). A probability value <0.05 was considered statistically significant.

## Results

### Impact of Conditioned Medium on Cardiac Fibroblast Gene Expressions

**Figure 2:**
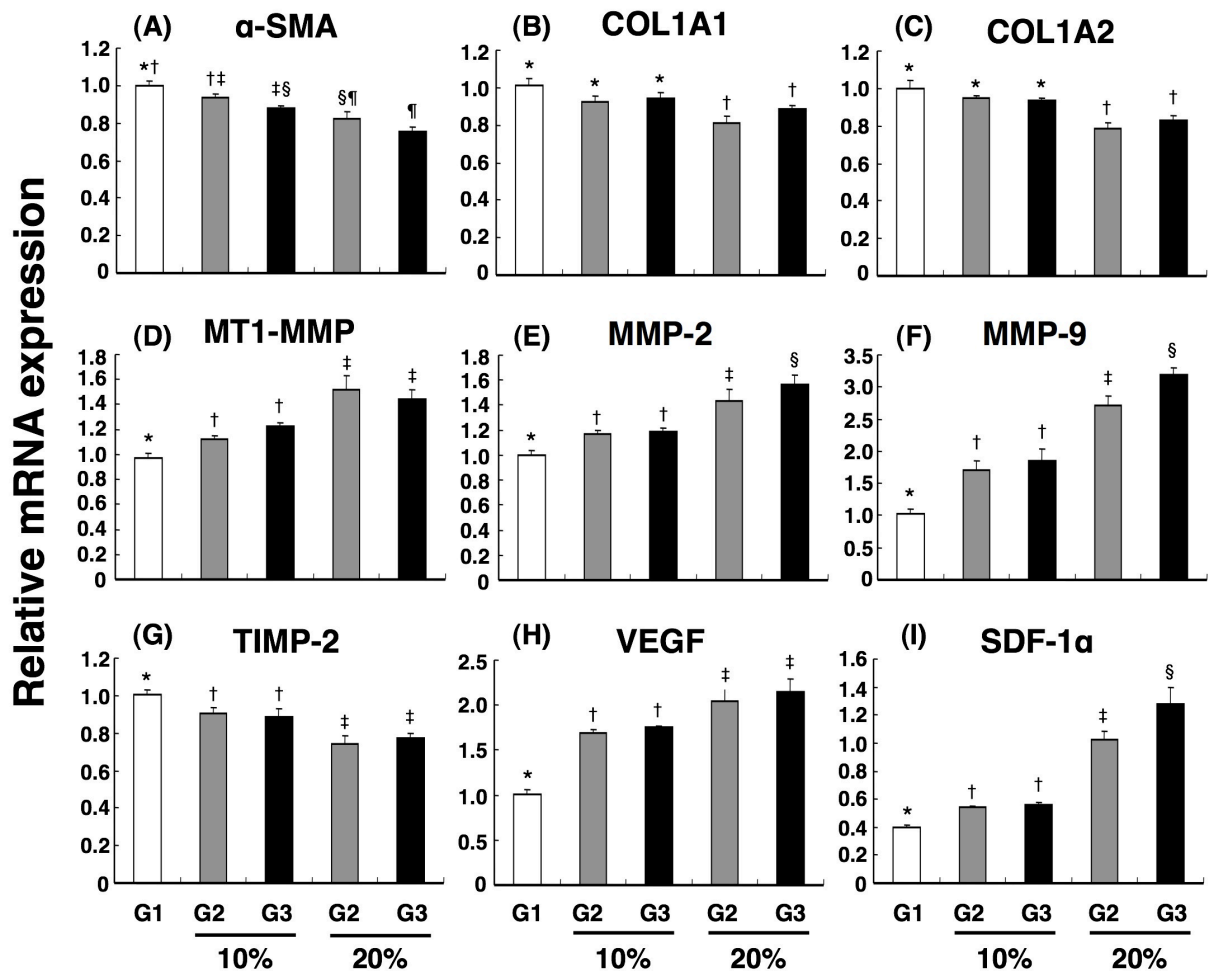
**Impact of conditioned medium on cardiac fibroblast gene expressions**. Effects of fresh medium (G1), 10% and 20% concentration of normal rat myocardium-derived conditioned medium (MDCM) (G2) and 10% and 20% of myocardial infarct-related MDCM (G3) on gene expressions of neonatal cardiac fibroblasts (n = 6 in each group). (A) mRNA expression of α-smooth muscle actin (SMA). G1 vs. G2 (10% & 20%) vs. G3 (10% & 20%), p < 0.01. Symbols (*, †, ‡, §, ¶) indicate significance (at 0.05 level) (by Bonferroni multiple comparison post hoc test). (B) & (C) mRNA expressions of both collagen type I α-1 (B) and collagen type I α-2 (C). *p < 0.02 between the indicated groups. (D) mRNA expression of major activator membrane type 1-matrix metalloproteinase (MT1-MMP). *p < 0.01 between the indicated groups. (E) & (F) mRNA expressions of matrix metalloproteinase (MMP)-2 and MMP-9. *p < 0.01 between the indicated groups. (G) mRNA expression of tissue inhibitor of metalloproteinase-2 (TIMP-2). *p < 0.02 between the indicated groups. (H) mRNA expressions of vascular endothelial growth factor (VEGF). *p < 0.01 between the indicated groups. (I) mRNA expressions of vascular endothelial growth factor (VEGF). *p < 0.001 between the indicated groups.

The mRNA expression of α-smooth muscle actin (α-SMA) (Figure 2A) in cultured CFBs was notably higher in Group 1 (CFBs cultured in fresh medium) than in Group 2 (CFBs co-cultured with normal MDCM) and Group 3 (CFBs co-cultured with infarct-related MDCM), and notably higher in Group 2 than in Group 3. On the other hand, the mRNA expressions of both collagen type I α-1 (Figure 2B) and collagen type I α-2 (Figure 2C) in cultured CFBs were similar between Group 1 and Group 2, whereas their expressions were notably suppressed in Group 3 compared with those in Group 1 and 2.

The mRNA expression of major activator membrane type 1-matrix metalloproteinase (MT1-MMP) (Figure 2D) in cultured CFBs was notably higher in Group 3 than in Group 1 and 2, and was significantly higher in Group 2 than in Group 1. In addition, the mRNA expressions of MMP-2 (Figure 2E) and MMP-9 (Figure 2F) in cultured CFBs were notably higher in Group 3 than in Group 1 and 2, and were remarkably higher in Group 2 than in Group 1. In contrast, the mRNA expression of tissue inhibitor of metalloproteinase-2 (TIMP-2) (Figure 2G) in cultured CFBs was notably lower in Group 3 than in Group 1 and 2, and was markedly lower in Group 2 than in Group 1.

The mRNA expression of VEGF (Figure 2H) in cultured CFBs was remarkably increased in Group 3 than in Group 1 and 2, and was significantly increased in Group 2 than in Group 1. Furthermore, the mRNA expression of SDF-1α (Figure 2I) in cultured CFBs was similar between Group 1 and Group 2, whereas it was notably increased in Group 3 than in the other groups.

### Impact of Conditioned Medium on Protein Expressions of Collagen Type I α-1 and α-SMA

**Figure 3:**
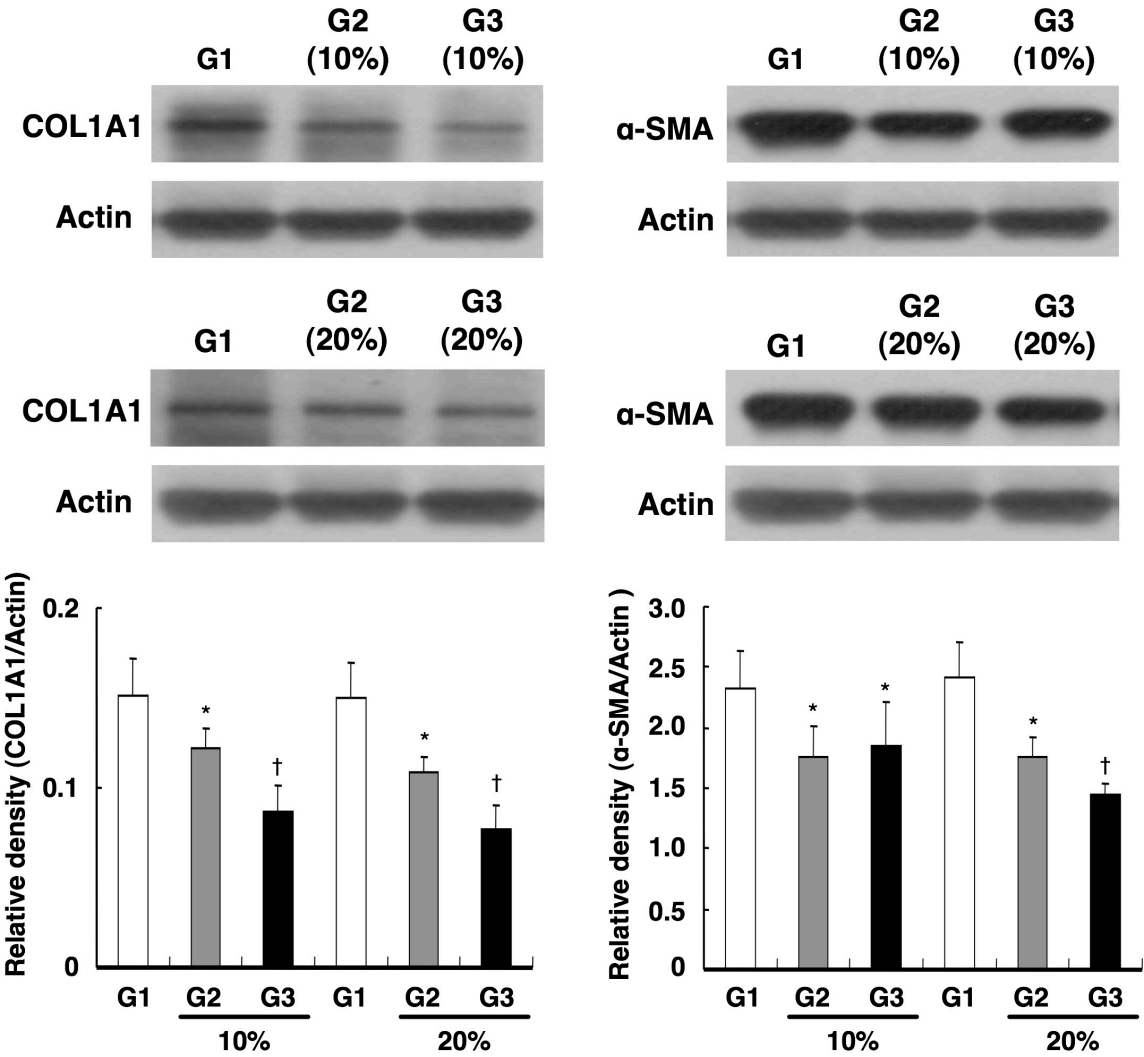
**Impact of conditioned medium on protein expressions of collagen type I α-1 and α-SMA**. (Left Panel) Protein expression of collagen type I α-1 (COL1A1) in cultured cardiac fibroblasts (CFBs) (n = 6 per group). *p = 0.002 between the indicated groups. Protein expression of COL1A1 in cultured CFBs. *p = 0.01 between the indicated groups. (Right Panel) Protein expression of α-smooth muscle actin (α-SMA) in cultured CFBs (n = 6 per group). G1 vs. 10% G2 vs. 10% G3, p = 0.031. G1 vs. 20% G2 vs. 20% G3, p = 0.003.

Western blot analysis demonstrated that the protein expression of collagen type I α-1 (Figure 3, left panel) in cultured CFBs was remarkably lower in Group 3 than in Group 1 and Group 2, and was significantly lower in Group 2 than in Group 1. Moreover, the α-SMA protein expression (Figure 3, right panel) in cultured CFBs was significantly suppressed in Group 3 than in the other two groups, but it did not differ between Group 1 and Group 2.

### Comparison of the Expressions of Gelatinolytic Activity of MMP-2 and MMP-9 in Supernatant of Cultured Neonatal Cardiac Fibroblasts

**Figure 4:**
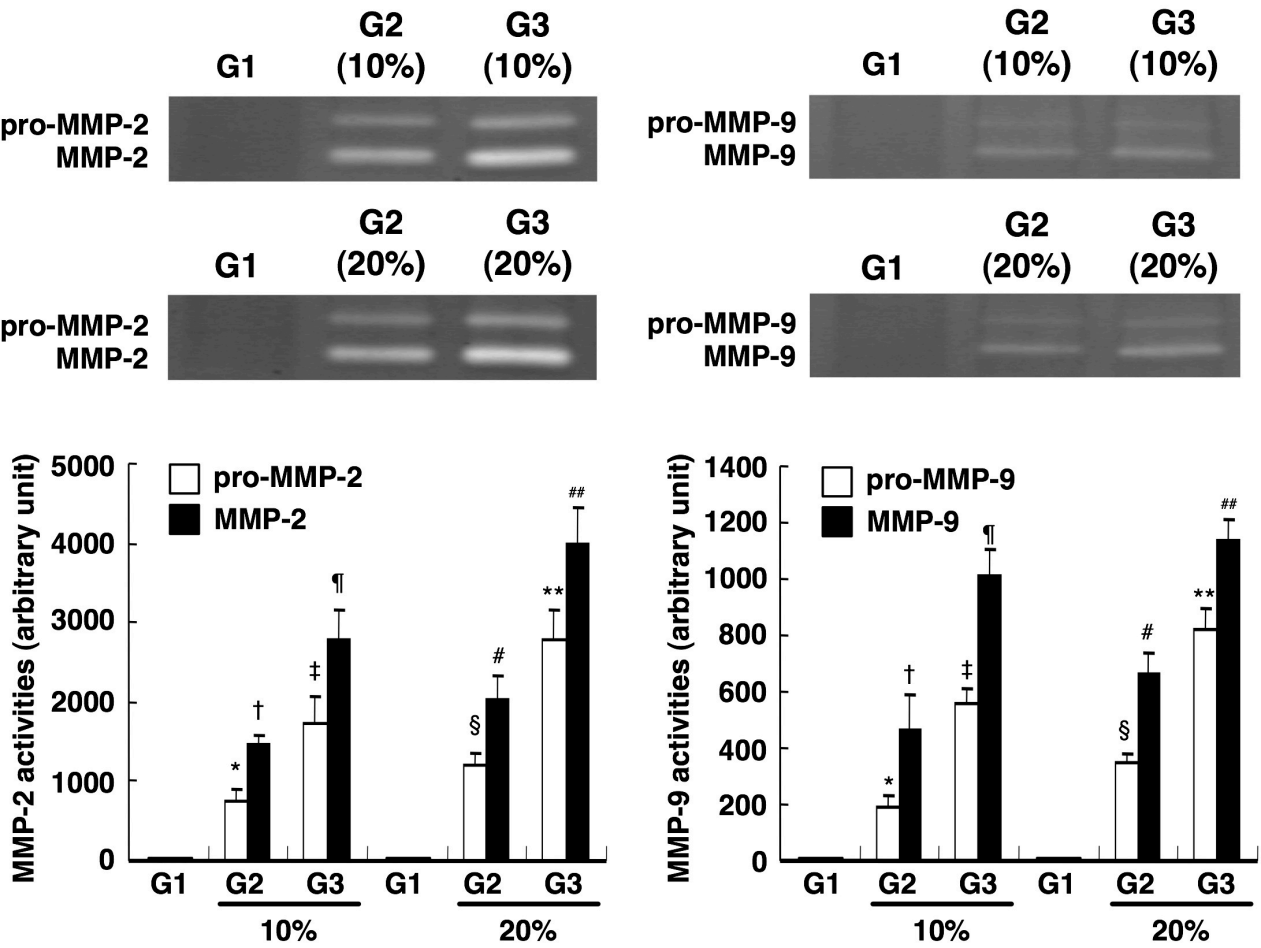
**Gelatinolytic activity of MMP-2 and MMP-9 in supernatant of cultured neonatal cardiac fibroblasts**. Expressions of supernatant gelatinolytic activity of MMP-2 and MMP-9 in fresh medium versus different conditioned media (n = 6 in each group). (Left Panel) Pro-MMP-2 and MMP-2 (cleaved). (1) G1 vs. 10% G2 vs. 10% G3, p < 0.0001 (* vs. ‡ or † vs. ¶, p < 0.001). (2) G1 vs. 20% G2 vs. 20% G3, p < 0.0001 (§ vs. ** or # vs. ##, p < 0.001). (Right Panel) Pro-MMP-9 and MMP-9 (cleaved). (1) G1 vs. 10% G2 vs. 10% G3, p < 0.0001 (* vs. ‡ or † vs. ¶, p < 0.001). (2) G1 vs. 20% G2 vs. 20% G3, p < 0.0001 (§ vs. ** or # vs. ##, p < 0.001).

The expressions of both pro-MMP-2 (pro-peptide) and active MMP-2 (cleaved) (Figure 4, left panel) were substantially increased in Group 3 compared with those in the other two groups, and were notably increased in Group 2 than in Group 1. Similarly, the expressions of both pro-MMP-9 (pro-peptide) and active MMP-9 (cleaved) showed consistent changes among the three groups (Figure 4, left panel).

### Increased Concentration of Interleukin (IL)-10, Transforming Growth Factor (TGF)-β, VEGF, SDF-1α and Basic Fibroblast Growth Factor (bFGF) in Infarct-related Conditioned Medium

**Figure 5:**
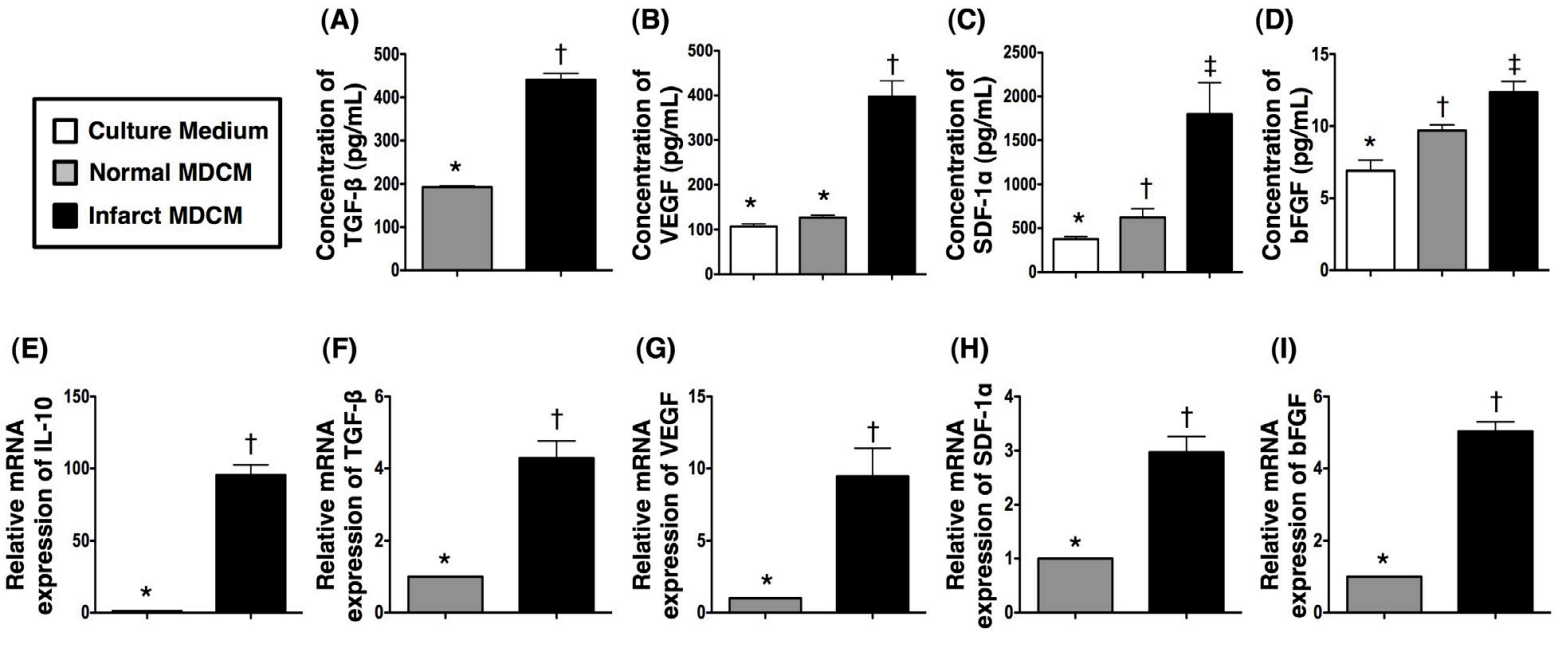
**ELISA analysis on conditioned medium and mRNA expression profile of cultured cellular components**. Comparison of ELISA findings of supernatant concentrations of transforming growth factor (TGF)-β, VEGF, stromal cell-derived factor (SDF)-1α, and basic fibroblast growth factor (bFGF) between normal MDCM and infarct-related MDCM after 36 h cell culture (n = 6 per group). (A) TGF-β, * vs. †, p < 0.001; (B) VEGF, *p < 0.0001 between the indicated groups; (C) SDF-1α, *p < 0.05 between the indicated groups; (D) bFGF, *p < 0.03 between the indicated groups. Comparisons of mRNA expressions of IL-10, TGF-β, VEGF, SDF-1α, and bFGF in normal cultured cardiac cell components and infarct-related cultured cell components after 36 h cell culture (n = 6 per group). (E) IL-10, * vs. †, p < 0.0001; (F) TGF-β, * vs. †, p = 0.0001; (G) VEGF, * vs. †, p = 0.0017; (H) SDF-1α, * vs. †, p < 0.0001; (I) bFGF, * vs. †, p < 0.0001.

To determine the trophic effects of the conditioned media, the concentrations of five most common and important chemokines (i.e. IL-10, TGF-β, VEGF, SDF-1α, and bFGF) were measured by ELISA (Figure 5, A-D). The concentration of IL-10 in normal MDCM was too low to be detected. The concentration of TGF-β in serum [i.e. fetal bovine serum (FBS)] of fresh medium was not measured because of its originally high concentration. As compared with normal MDCM, the concentration of TGF-β was remarkably higher in infarct-related MDCM. The concentration of VEGF did not differ between fresh medium and normal MDCM, whereas it was significantly higher in infarct-related MDCM compared with both fresh medium and normal MDCM. The concentrations of SDF-1α and bFGF were notably higher in normal MDCM and infarct-related MDCM than in fresh medium, and significantly higher in infarct-related MDCM than in normal MDCM.

### Increased mRNA Expression of IL-10, TGF-β, VEGF, SDF-1α, and bFGF in 36-hour cultured myocardium components

To determine whether the trophic effects of chemokines in conditioned medium were derived from cultured cellular components, the mRNA expressions of IL-10, TGF-β, VEGF, SDF-1α, and bFGF (Figure 5, E-I) were measured in this study. The mRNA expressions of IL-10 and TGF-β, two indicators of anti-inflammation, were remarkably higher in infarct-related cultured cellular components than in normal cultured cellular components. Besides, the mRNA expressions of VEGF, SDF-1α, and bFGF, three pro-angiogenic indexes, were substantially higher in infarct-related cultured cellular components than in normal cultured cellular components.

### Impact of Conditioned Medium Treatment on 90-Day Left Ventricular Function and Fractional Shortening

The initial left ventricular ejection fraction (LVEF), fractional shortening (FS), LVEDD and LVESD were similar among the six groups (Table 1). Besides, there was also no significant difference between the 90-day LVEF and FS among Group I, II and III. However, the 90-day LVEF and FS were remarkably lower, whereas the LVEDD and LVESD were notably higher in Group IV, V, and VI than in Group I, II, and III. Furthermore, the 90-day LVEF and FS were significantly lower in Group IV than in Group V and VI, and notably lower in Group V than in Group VI. Moreover, the 90-day LVEDD and LVESD were significantly higher in Group IV than in Group V and Group VI, and notably higher in Group V than in Group VI. These findings imply that conditioned media, especially those derived from the infarcted heart, was effective in preserving LV function and inhibiting LV remodeling after AMI.

**Table 1:** Echocardiographic Findings Prior to and on Day 90 after AMI

Variables	Group I (n = 6)	Group II (n = 6)	Group III (n = 6)	Group IV (n = 6)	Group V (n = 6)	Group VI (n = 6)	P‡ value
LVEF (%)*	81.5 ± 2.07	80.8 ± 1.39	79.7 ± 1.48	80.8 ± 1.44	81.7 ± 3.18	80.2 ± 1.75	0.512
FS (%)*	42.4 ± 1.95	43.7 ± 2.46	42.9 ± 1.26	43.2 ± 1.82	44.2 ± 1.88	43.6 ± 1.61	0.648
LVEDD (cm)*	0.60 ± 0.01	0.61 ± 0.01	0.62 ± 0.02	0.60 ± 0.02	0.59 ± 0.03	0.60 ± 0.01	0.871
LVESD (cm)*	0.33 ± 0.02	0.32 ± 0.01	0.34 ± 0.01	0.32 ± 0.02	0.31 ± 0.03	0.33 ± 0.01	0.794
LVEF (%)†	79.8^a ^± 1.46	79.3^a ^± 2.44	79.2^a ^± 2.07	63.4^b ^± 1.71	69.8^c ^± 2.03	74.8^d ^± 2.87	<0.0001
FS (%)†	43.0^a ^± 1.21	43.2^a ^± 1.75	43.1^a ^± 0.85	30.9^b ^± 0.50	35.2^c ^± 2.19	38.7^d ^± 1.21	<0.0001
LVEDD (cm)†	0.61 ± 0.01^a^	0.60 ± 0.01^a^	0.59 ± 0.02^a^	1.0 ± 0.01^b^	0.77 ± 0.02^c^	0.69 ± 0.03^d^	<0.0001
LVESD (cm)†	0.34 ± 0.01^a^	0.31 ± 0.02^a^	0.33 ± 0.02^a^	0.66 ± 0.02^b^	0.49 ± 0.02^c^	0.40 ± 0.02^d^	<0.0001

Data expressed as means ± SD.

AMI = acute myocardial infarction; LVEF = left ventricular ejection fraction; FS = fractional shortening; LVEDD = left ventricular end-diastolic dimension; LVESD = left ventricular systolic dimension.

* Transthoracic echocardiography performed at day 0 prior to AMI induction.

† Transthoracic echocardiography performed on day 90 after AMI induction.

Group I = sham control treated by fresh medium;

Group II = sham control treated by normal heart myocardium-derived conditioned medium (MDCM);

Group III = sham control treated by infarcted-related MDCM;

Group IV = AMI induction treated by fresh medium;

Group V = AMI induction treated by normal heart MDCM;

Group VI = AMI induction treated by infarcted-related MDCM.

‡ One-way ANOVA on the arcsine transformed data was used to improve the normality for statistical analysis. Letters (^a, b, c, d^) indicate significance (at 0.05 level) by Bonferroni multiple comparison post hoc test (^a ^versus ^b, c, d^, ^b ^versus ^c, d^, ^c ^versus ^d^, all p values <0.05)

### Impact of Conditioned Medium Treatment on Regulating mRNA Expressions of SDF-1α, VEGF, Endothelial Nitric Oxide Synthase (eNOS), Bcl-2, Bax, and Caspase-3 in LV Myocardium

**Figure 6:**
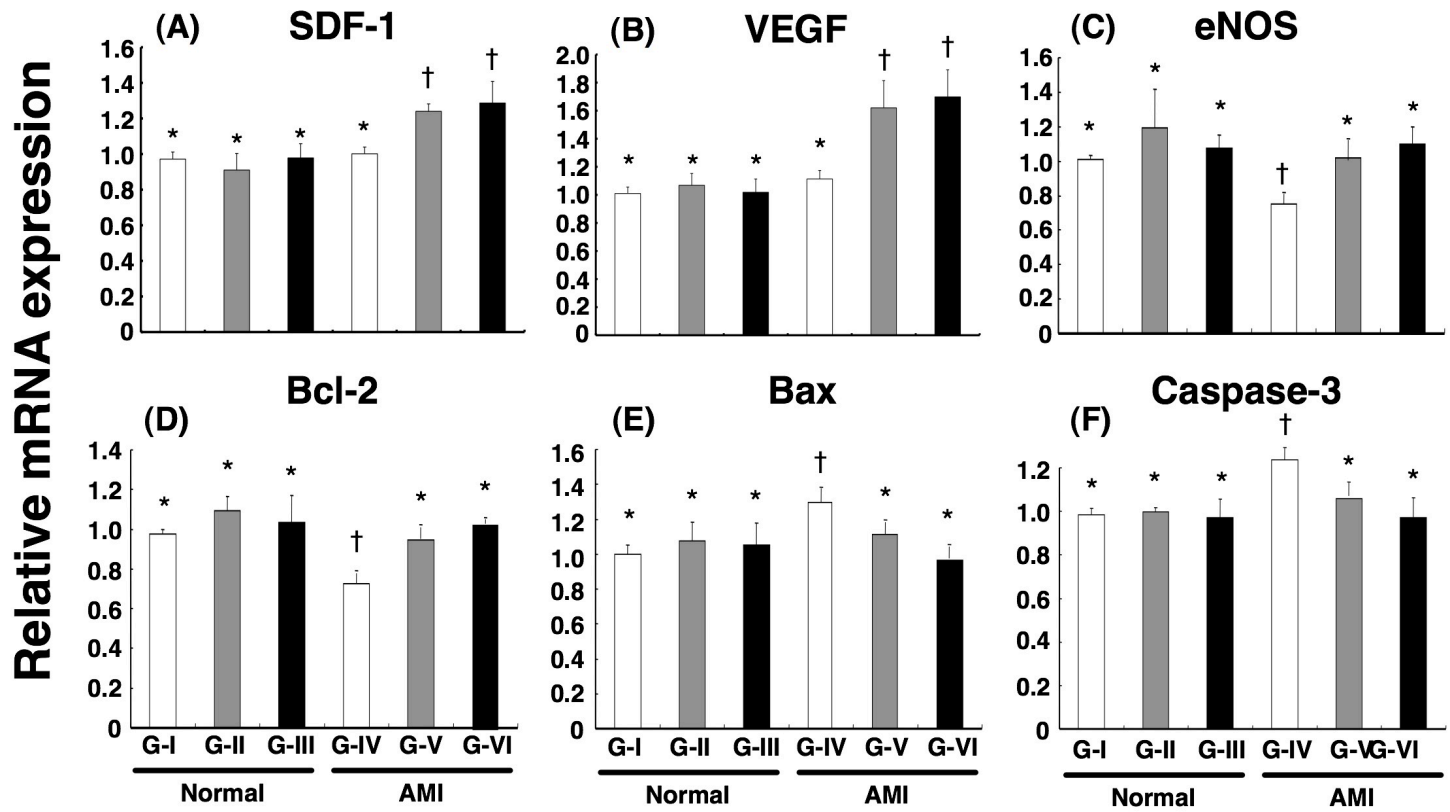
**Impact of conditioned medium treatment on mRNA expression of angiogenic and apoptotic factors in left ventricular myocardium**. Real-time PCR showing significantly lower mRNA expressions of (A)SDF-1α, (B)VEGF, (C) endothelial nitric oxide synthase (eNOS), and (D) Bcl-2 in LV myocardium in Group IV (AMI treated by fresh medium) than in other groups (p < 0.03) (n = 6 in each group). Note also remarkably higher gene expressions of (E) Bax and (F) caspase-3 in Group IV than in other groups (p < 0.01).

The impact of conditioned medium treatment on 90-day left ventricular function and fractional shortening is shown in Table 1. Real-time PCR analyses showed remarkably lower mRNA expressions of SDF-1α, VEGF, eNOS and Bcl-2 in Group IV than in other groups (Figure 6). Conversely, the mRNA expressions of Bax and caspase 3 were notably higher in Group IV than in other groups. These findings suggest that conditioned medium therapy up-regulated chemokines for angiogenesis and suppressed cellular apoptosis in LV myocardium.

### Impact of Conditioned Medium Treatment on Oxidative Stress

**Figure 7:**
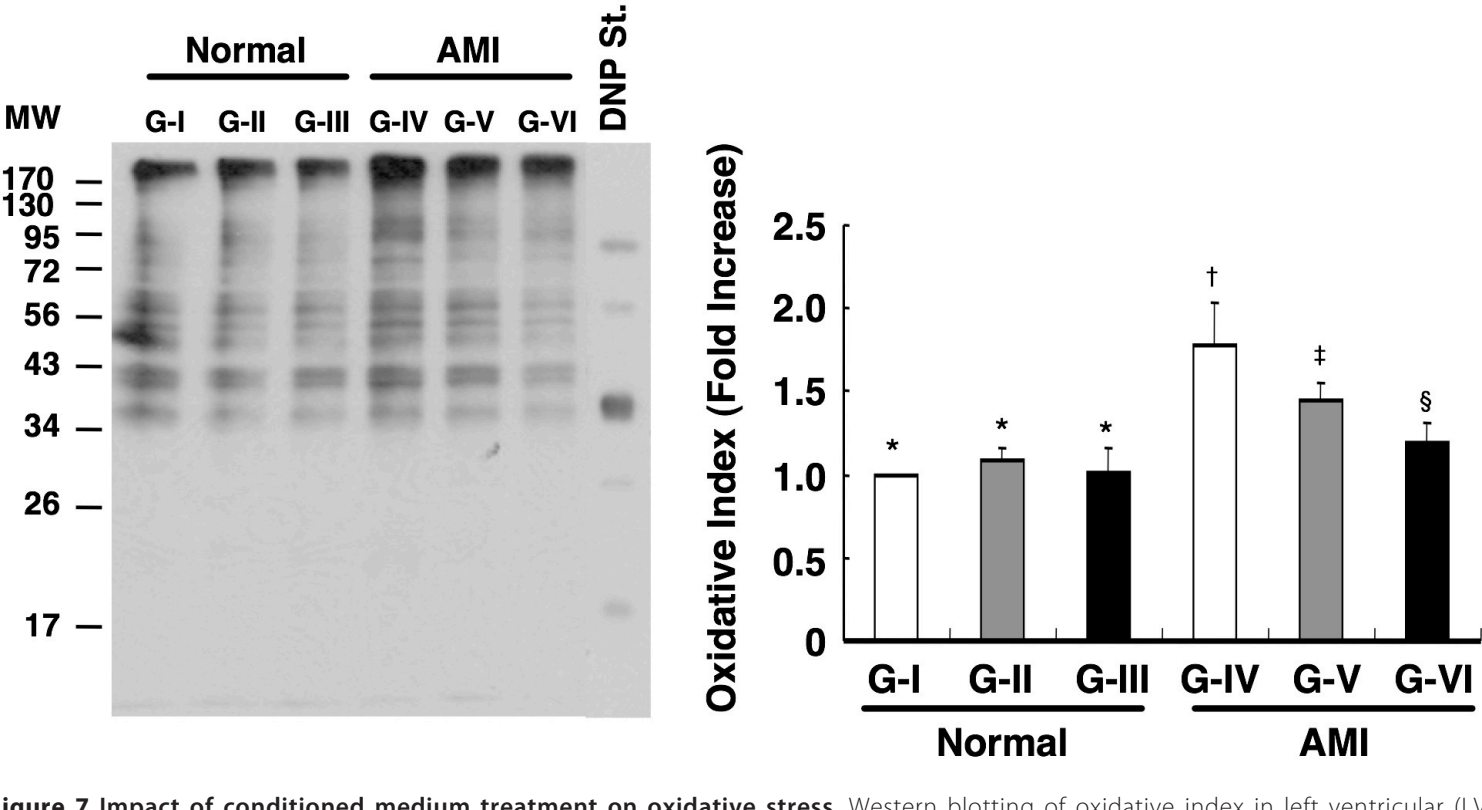
**Impact of conditioned medium treatment on oxidative stress**. Western blotting of oxidative index in left ventricular (LV) myocardium of Group I to VI on day 90 after AMI induction (left), with quantification results of each group (n = 6) shown (right). *p < 0.003 between the indicated groups.

Western blotting revealed that although the mitochondrial oxidative stress in LV myocardium did not differ among Group I, II, and III on day 90 after AMI induction, it was significantly higher in Group IV than in other groups and was notably higher in Group V than in Group VI (Figure 7). The results, therefore, showed an increase in oxidative stress after AMI that was significantly suppressed by MDCM, especially infarct-related MDCM.

### Impact of Conditioned Medium Treatment on Enhancing Protein Expressions of Cx43, CXCR4, and SDF-1α

**Figure 8:**
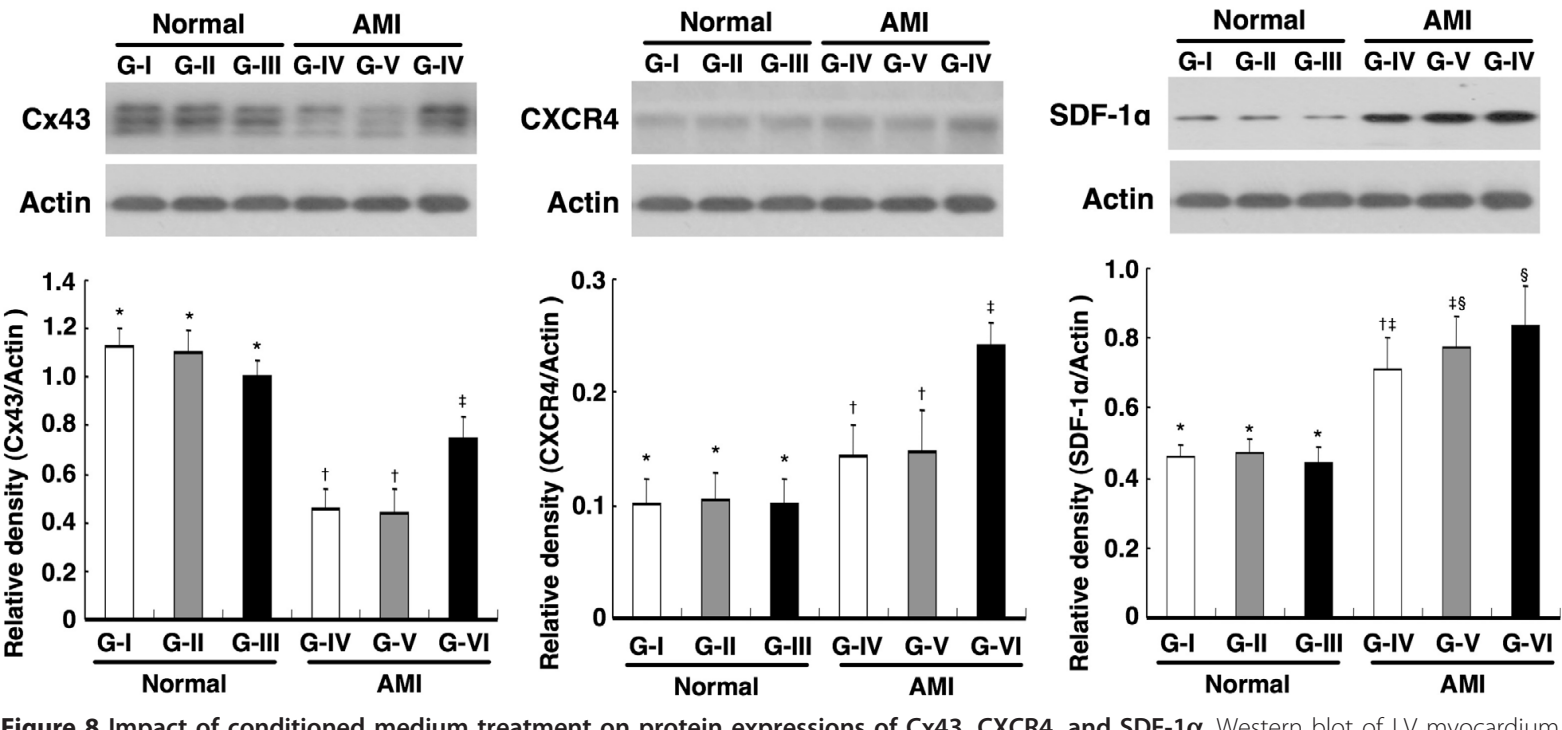
**Impact of conditioned medium treatment on protein expressions of Cx43, CXCR4, and SDF-1α**. Western blot of LV myocardium (n = 6 in each group). (Left) Protein expression of connexin43 (Cx43). *p < 0.0001 between the indicated groups. (Middle) Protein expression of CXCR4. *p < 0.001 between the indicated groups. (Right) Protein expression of SDF-1α. *p < 0.001 between the indicated groups.

Cx43 protein expression in LV myocardium on day 90 after AMI induction was similar among Group I, II, and III, and was also similar between Group IV and Group V (Figure 8, left panel). On the other hand, the expression was markedly higher in Group I, II, and III than in Group IV, V, and VI, and notably higher in Group VI than in Group IV and V. The results, therefore, demonstrated a notable suppression in Cx43 expression after AMI induction. The expression, however, was significantly restored after administration of infarct-related MDCM.

CXCR4 protein expression in LV myocardium on day 90 after AMI induction did not differ among Group I, II, and III was also similar between Group IV and V (Figure 8, middle panel). However, the expression was significantly higher in Group IV, V, and VI than in Group I, II, and III, and was significantly higher in Group VI than in Group IV and V.

In addition, there was also no significant difference in SDF-1α protein expression in LV myocardium among Group I, II and III and among Group IV, V and VI on day 90 after AMI (Figure 8, right panel). However, the expression was significantly higher in Group IV, V, and VI than in Group I, II and III.

### Impact of Conditioned Medium on Number of von Willebrand Factor (vWF)-Positive Cells

**Figure 9:**
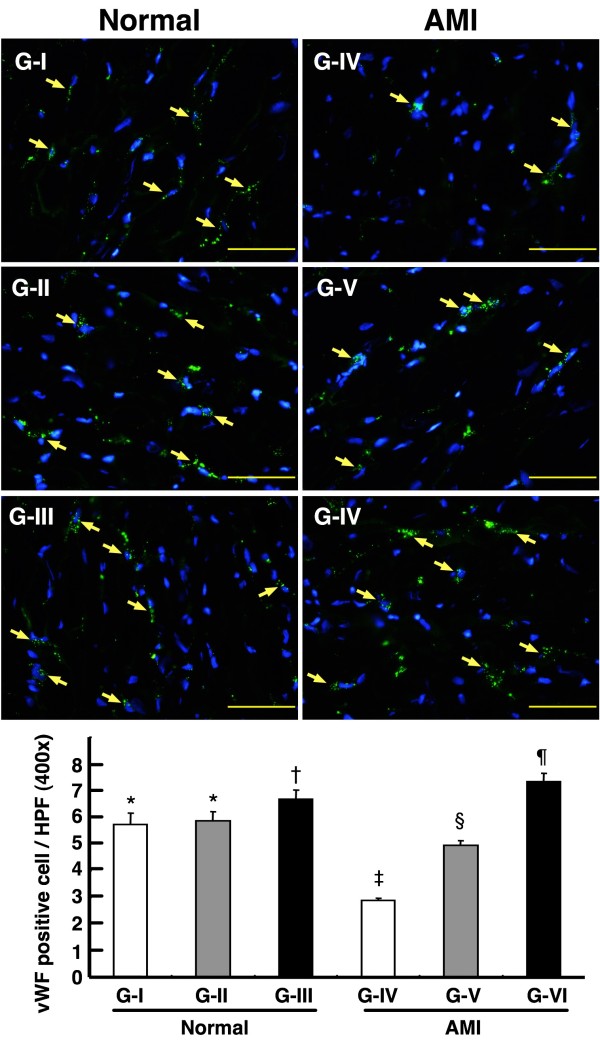
**Impact of conditioned medium on number of von Willebrand factor (vWF)-positive cells**. Immunofluorescent staining (400×) for von Willebrand factor (vWF)-positive cells in LV myocardium in sham-operated controls and infarcted animals (n = 6 in each group). *p < 0.001 between the indicated groups. Scale bars in right lower corner represent 50 μm.

Immunofluorescent staining identified remarkably higher number of vWF-positive cells, a marker of endothelial cells, in Group VI than in other groups (Figure 9). The number was also significantly higher in Group I, II, and III than in Group IV and V, and also notably higher in Group V than in Group IV. However, it showed no difference among Group I, II, and III. These findings indicate that treatment with infarct-related MDCM had a positive impact on angiogenesis.

### Impact of Conditioned Medium on Cellular Proliferation in Infarct Area of Left Ventricle

**Figure 10:**
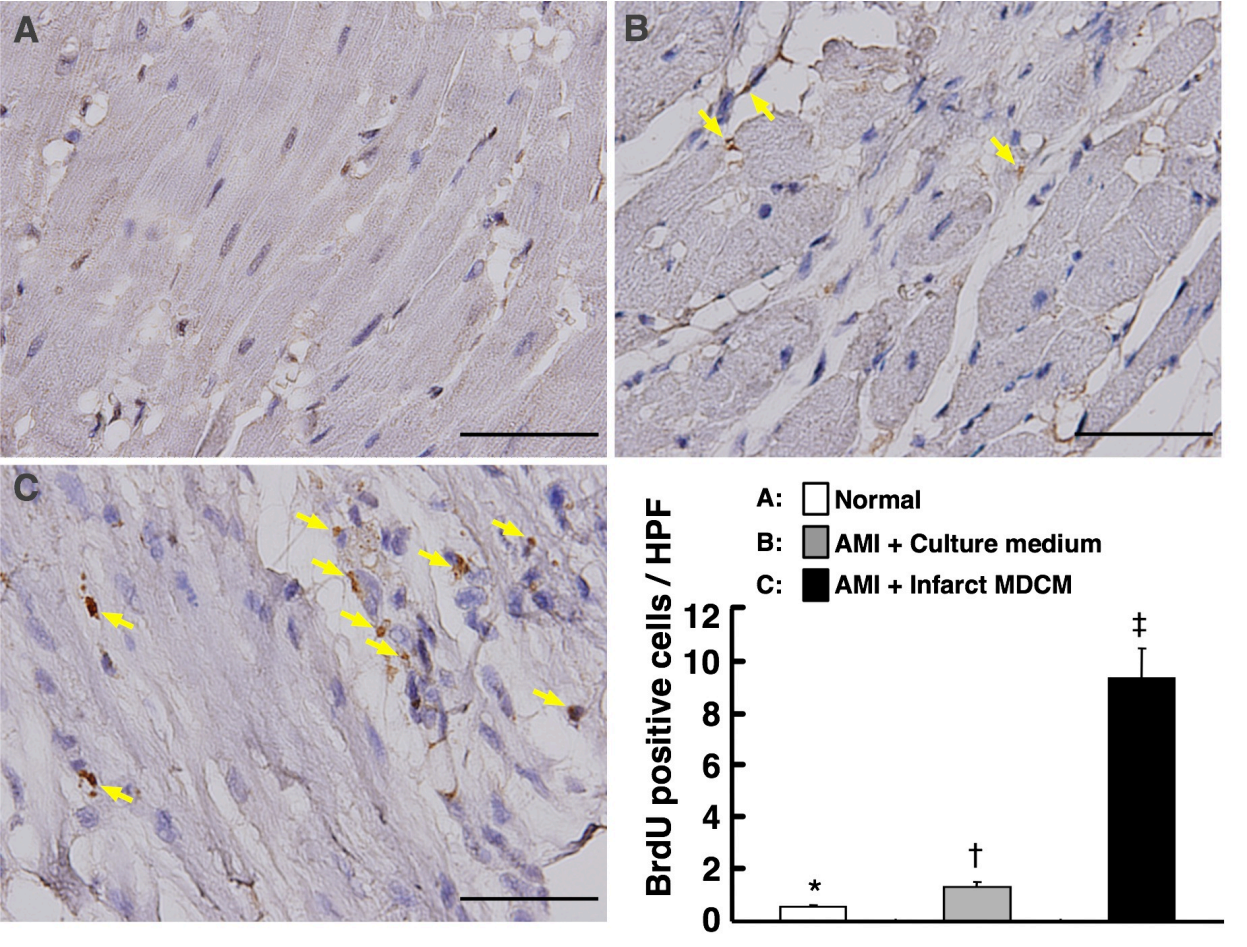
**Impact of conditioned medium on cellular proliferation in infarct area of left ventricle**. Immunohistochemical (IHC) staining (400×) for the distribution of proliferative cells in infarction area of LV myocardium (n = 6 in each group). *p < 0.0001 between the indicated groups. Scale bars in right lower corner represent 50 μm.

To determine whether conditioned medium treatment enhanced cellular proliferation in LV infarct area, intra-venous injection of BrdU was given to Group I, IV, and VI. The results demonstrated that by day 90 after AMI induction, the cellular uptake of BrdU, an index of cellular proliferation, was remarkably elevated in Group VI compared with that in other groups (Figure 10). It was also significantly higher in Group IV than in Group I.

### Impact of Conditioned Medium on Reducing Collagen Expression

**Figure 11:**
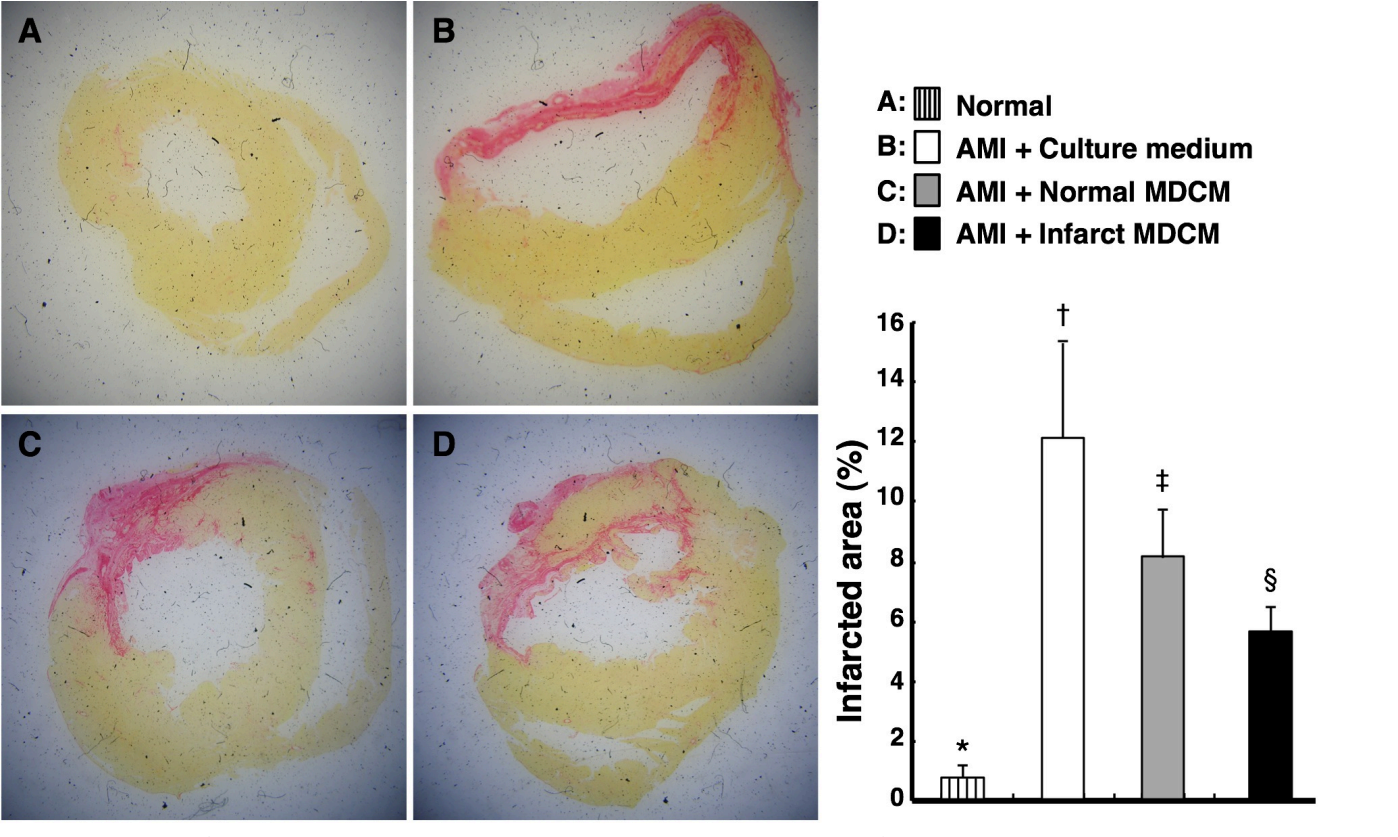
**Impact of conditioned medium on collagen expression**. Sirius red staining for collagen deposition in LV myocardium (n = 4). *p < 0.001 between the indicated groups.

To investigate whether conditioned medium treatment reduced collagen expression in infarct area of LV myocardium, Sirius red staining was performed for Group I, IV, V, and VI in the current study. The collagen deposition area was substantially higher in Group IV than in other groups. It was also remarkably higher in Group V than in Group VI and I, and was significantly higher in Group VI than in Group I (Figure 11). These findings suggest that treatment with infarct-related MDCM significantly inhibited collagen deposition in infarct zone of LV myocardium.

### Impact of Conditioned Medium on Angiogenesis

**Figure 12:**
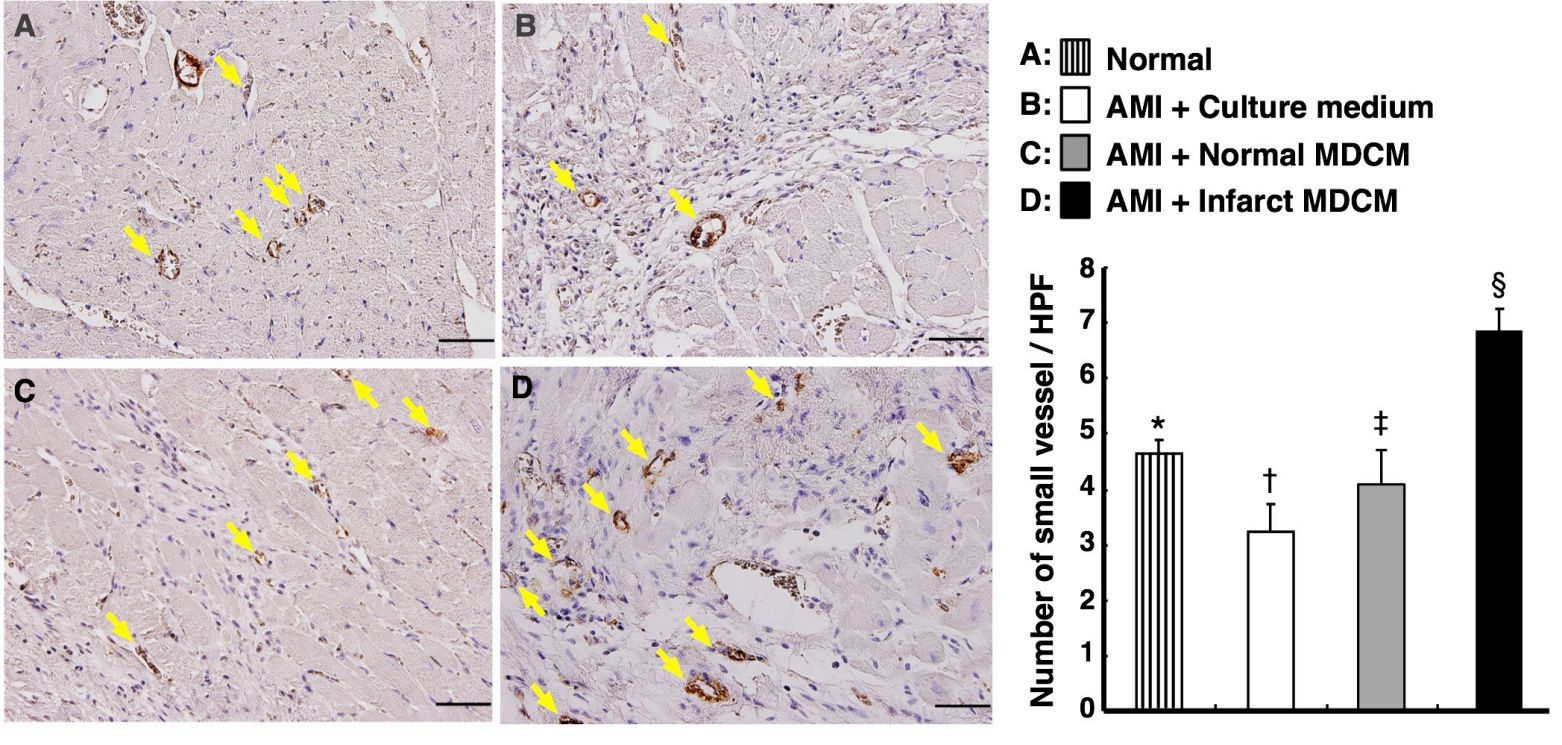
**Impact of conditioned medium on angiogenesis**. The number of arterioles in infarct LV myocardium (n = 6). Quantification (right panel) of small vessels (diameters ≤15 mm) (yellow arrows) on 90 day following AMI induction (200 ×). *p < 0.0001 between the indicated groups. Scale bars in right lower corner represent 50 μm.

IHC staining for Group I, IV, V and VI demonstrated notably higher number of small vessels positively stained for α-SMA in Group VI than in other groups (Figure 12). The number was also remarkably higher in Group I than in Group IV and V, and was notably increased in Group V than in Group IV. These findings indicate that treatment with infarct-related MDCM significantly enhanced neovascularization.

## Discussion

The present study, which investigated the potential impact of MDCM on heart function and LV remodeling in a rat AMI model, provided several valuable implications. First, the gene expression of collagen and the protein expressions of both the collagen and α-SMA of CFBs were significantly suppressed after co-culturing with infarct-related MDCM. Second, gelatinolytic analysis demonstrated notably increased MMP-2 and MMP-9 activities in CFBs after co-culturing with infarct-related MDCM. Third, ELISA finding showed remarkably higher VEGF, SDF-1α, bFGF, and TGF-β levels in infarct-related MDCM compared with those in normal MDCM. Fourth, fibrosis and oxidative stress in LV myocardium were markedly attenuated, whereas CXCR4 and SDF-1α protein expressions as well as angiogenesis/vasculogenesis were substantially increased after treatment with infarct-related MDCM on day 90 after AMI. Importantly, both LVEF and FS were notably preserved and LV remodeling was remarkably suppressed following infarct-related MDCM administration.

### Conditioned Medium Treatment Improved LV Function after AMI

Although stem cell therapy appears to be an attractive and promising option in treatment of ischemic organ dysfunction [1-6,8-10], the principal mechanism is still poorly defined [3-5,8,9]. Growing evidence suggests that the reparation, regeneration, and improvement in ischemic organ dysfunction after stem cell therapy is mainly due to its cytokine/paracrine [3-5,10,11,13] effects and angiogenesis [3-5,8,9] rather than the results of differentiation of transplanted cells per sec into particular cell phenotype. Indeed, studies have revealed that MSC-derived conditioned medium significantly contributes to the positive impacts of cell therapy [13,22]. Interestingly, while the conditioned medium derived from MCSs has been well reported to preserve the function of other ischemia-related organ disorders [13,22,23], the therapeutic benefit of MDCM in ischemia-related LV dysfunction has not been reported. The novel finding in the present study is that infarct-related MDCM notably preserved heart function and markedly attenuated LV remodeling after AMI. Furthermore, although it is less effective compared with infarct-related MDCM, normal MDCM treatment still significantly improved heart function after AMI. Therefore, our findings, in addition to strengthening those of previous studies [13,22,23], further highlight the therapeutic potential of conditioned medium derived from myocardial components of ischemic heart, a mimicked ischemic preconditioning, in the treatment of ischemic heart disease.

Interestingly, previous clinical observational studies [3,21] have shown that patients with ischemic preconditioning experience less myocardial damage, better preservation of LV function, and more favorable clinical outcome after AMI compared with those without. Consistently, numerous animal model studies [22,23] have also established a therapeutic benefit of preconditioning in preventing myocardial damage from ischemia-reperfusion injury. Although the precise mechanisms of preconditioning against myocardial damage from AMI attack or ischemia-reperfusion injury are till not fully understood, this phenomenon may at least partly account for the positive therapeutic impact of treatment with infarct-related MDCM on LV function in the current study.

### Possible Mechanisms Underlying MSC-Derived Conditioned Medium Therapy in Improving Heart Function

The paracrine mediators secreted by MSCs have been identified to be chemokines and cytokines in both the cultured medium and MSC-implanted area [11,13,22,24]. The chemokines, which consist mainly of SDF-1α, VEGF, and HGF, are called trophic factors that have been reported to contribute to the mobilization of endothelial progenitor cell/MSC into circulation and homing to ischemic area for angiogenesis/vasculogenesis and regeneration, thereby improving ischemia-related organ dysfunction [24-27]. In addition, cytokines including MMP-2, MMP-9, and TIMP, which are well known regulators of extra-cellular matrix (ECM) formation [13,28], have also been shown to modulate CFB activity and play an essential role in regulating LV remodeling [13]. Indeed, previous studies have already demonstrated the importance of trophic mediators in this process [11,13,22,24-28].

### Improvement of Heart Function after AMI from Findings of Current Study--Paracrine Effects and Angiogenesis

One important finding in the current study is that ELISA showed a remarkably higher level of VEGF, a common index of angiogenesis, and SDF-1α, a well-known trophic chemokine, in infarct-related MDCM compared with normal MDCM and fresh medium. Moreover, real-time PCR showed that the mRNA expressions of VEGF and SDF-1α in both cultured CFBs and infarcted LV myocardium were significantly higher using normal MDCM compared with fresh medium. The expressions of these mediators, interestingly, were further enhanced when infarct-related MDCM was used. Furthermore, Western blot analysis demonstrated a notable increase in CXCR4 protein expression, a marker of endothelial progenitor cells, in infarcted LV myocardium when normal MDCM was used instead of fresh medium. It was further upregulated after administration of infarct-related MDCM. Moreover, the protein expression of SDF-1α, a chemokine for EPC mobilization, was also elevated in infarcted LV after administration of infarct-related MDCM compared with infusion of fresh medium. Finally, real-time PCR and Immunofluorescent staining of infarcted LV myocardium showed that the expression of eNOS, an indicator of endothelial function, and the number of vWF-positive cells, a marker of endothelial cells, were significantly higher when normal MDCM was applied and further elevated when infarct-related MDCM was given as compared with fresh medium. The results, in addition to strengthening those of previous studies [24-27], are consistent with other findings in this study including an increase in the positivity of α-SMA staining (i.e. an indicator of angiogenesis/vasculogenesis) and cellular proliferation in infarcted LV myocardium. Taken together, our findings could, at least in part, account for the preservation of cardiac function in the setting of AMI after MDCM treatment.

Interestingly, recent studies have shown that gene therapy using over-expressions of VEGF and SDF-1 genes significantly improves ischemia-related LV dysfunction in experimental studies [29,30]. Similarly, results of the current study using myocardial infarction-induced enhancement of paracrine secretions for treatment of AMI, in addition to being comparable to those of the recent studies, further clarify the roles of chemokine/cytokine and the mechanisms underlying the improvement in heart function after AMI.

### Inhibition of LV Remodelling--Crucial Role of MMPs

The principal finding in the present study is that both conditioned media enhanced the mRNA expressions of MMP-2, MMP-9, and TM1-MMP in cultured CFBs. In contrast, TIMP-2 mRNA expression in cultured CFBs, an indicator of the trend of developing cardiac fibrosis, was markedly suppressed by both conditioned media. Additionally, the gelatinolytic activities of MMP-2 and MMP-9 in supernatant of cultured CFBs were remarkably upregulated by both conditioned media. On the other hand, α-SMA expression and collagen secretion by cultured CFBs were remarkably suppressed by conditioned media. Furthermore, Sirius-red staining showed that the fibrosis in LV infarct area was significantly reduced by normal MDCM and further suppressed by infarct-related MDCM as compared with fresh medium. Our findings, therefore, in addition to reinforcing the results of previous studies [13], may partially explain the attenuation of post-AMI LV remodeling after MDCM treatment.

### Impact of Conditioned Medium on Oxidative Stress, Cellular Apoptosis, and Cx43 expression

The mRNA expressions of Bax and caspase-3, indexes of apoptosis, were notably reduced in infarcted LV myocardium after treatment with either conditioned medium compared with fresh medium. On the other hand, the expressions of Bcl-2 and eNOS, two indicators of anti-apoptosis, were significantly elevated in infarcted LV myocardium following administration of the two types of conditioned media compared to fresh medium treatment. Besides, Western blot demonstrated remarkably reduced oxidative stress in infarcted LV myocardium after administration of normal MDCM compared to treatment with fresh medium. It was further suppressed in infarct-related medium. The link between increased oxidative stress and cellular apoptosis has been established in ischemic condition [4,5,31]. Furthermore, an association between an increase in both cellular apoptosis and oxidative stress and a decreased Cx43 expression in ischemic myocardium, which plays a key role in electrical coupling between cardiomyocytes [32,33], has been demonstrated in our previous studies [4,5]. The notable reduction in protein expression of Cx43 in infarcted LV myocardium and its restoration after administration of infarct-related MDCM further support our findings of less LV remodeling and better LV function in animals receiving infarct-related MDCM compared with the other treatment groups.

### Study Limitations

This study has limitations. First, the harvested cellular elements from ex vivo digestion contain various cellular components including cardiomyocytes and CFBs that together constitute 90% of cells in myocardium and also endothelial cells that make up less than 10% of the cell population. Therefore, although both chemokines and cytokines were identified in MDCM, this study cannot specifically identify their exact sources. Second, since a variety of complex cytokine-mediated interactions after myocardial injury have been suggested [34], other mediators that may participate in the process of post-AMI LV remodeling cannot be identified without a detailed proteomic screening study for MDCM. Third, since the heart has been suggested to contain endogenous cardiac stem cells [35], their precise involvement in tissue regeneration and repair after MDCM treatment remains unknown. Finally, although studies have previously reported that myocardium-derived medium can induce the differentiation of bone marrow mesenchymal stem cells [36], the current study did not evaluate the impact of MDCM on the differentiation of the stem cells to provide information to address this issue.

In conclusion, although the exact mechanisms underlying the positive therapeutic potential of MDCM treatment in suppressing LV remodeling and preserving LV function after AMI remain uncertain, our demonstration of further enhancement of the therapeutic effect using infarct-related conditioned medium suggests that an interplay of cytokines, a reduction in oxidative stress, an enhanced stem cell homing effect and angiogenesis appear to be the key elements contributing to the improvement in heart function after infarction. These findings also support the proposal that the positive impact of MSC therapy on ischemia-related heart dysfunction is due to its paracrine effects instead of differentiation of implanted MSCs into specific cell phenotype in the ischemic area.

## Competing interests

The authors declare that they have no competing interests.

## Author's information

Cheuk-Kwan Sun contributed equally as the first author to this work. Morgan Fu contributed equally compared with the corresponding author to this work.

## Authors' contributions

All authors have read and approved the final manuscript. SL, YHK, YCL, and CKS designed the experiment, drafted and performed animal experiments. LTC, THT, SC, KHY, and CJW were responsible for the laboratory assay and troubleshooting. MF and HKY participated in refinement of experiment protocol and coordination and helped in drafting the manuscript.
